# Prostaglandin F_2α_-F-prostanoid receptor regulates CXCL8 expression in endometrial adenocarcinoma cells via the calcium–calcineurin–NFAT pathway

**DOI:** 10.1016/j.bbamcr.2009.09.018

**Published:** 2009-12

**Authors:** Kurt J. Sales, David Maldonado-Pérez, Vivien Grant, Rob D. Catalano, Martin R. Wilson, Pamela Brown, Alistair R.W. Williams, Richard A. Anderson, E. Aubrey Thompson, Henry N. Jabbour

**Affiliations:** a MRC Human Reproductive Sciences Unit, The Queen's Medical Research Institute, 47 Little France Crescent, The University of Edinburgh, Edinburgh, EH16 4TJ, UK; b Reproductive and Developmental Sciences, The Queen's Medical Research Institute, The University of Edinburgh, Edinburgh, EH16 4TJ, UK; c Pathology, The Queen's Medical Research Institute, The University of Edinburgh, Edinburgh, EH16 4TJ, UK; d Department of Cancer Biology, Mayo Clinic Comprehensive Cancer Center, Jacksonville, Florida 32224, USA

**Keywords:** PGF_2α_, FP receptor, Chemokine, Calcineurin, Prostaglandin, CXCL8

## Abstract

Pro-inflammatory mediators, like prostaglandin (PG) and chemokines, promote tumourigenesis by enhancing cell proliferation, migration of immune cells and recruitment of blood vessels. Recently we showed elevated expression of the chemokine (C-X-C motif) receptor 2 (CXCR2) in endometrial adenocarcinomas localized to neutrophils and neoplastic epithelial and vascular cells. Furthermore we found that PGF_2α_-F-prostanoid (FP) receptor regulates the expression of the CXCR2 ligand CXCL1, to promote neutrophil chemotaxis in endometrial adenocarcinomas. In the present study we identified another CXCR2 ligand, CXCL8 as a target for PGF_2α_-FP receptor signalling which enhances epithelial cell proliferation in endometrial adenocarcinoma cells *in vitro* and in nude mice *in vivo*. We found that PGF_2α_-FP receptor interaction induces CXCL8 expression in endometrial adenocarcinoma cells via the protein kinase C–calcium–calcineurin–NFAT signaling pathway. Promoter analysis revealed that CXCL8 transcriptional activation by PGF_2α_ signaling is mediated by cooperative interactions between the AP1 and NFAT binding sites. Furthermore, PGF_2α_ via the FP receptor induced the expression of the regulator of calcineurin 1 isoform 4 (RCAN1-4) via the calcineurin/NFAT pathway in a reciprocal manner to CXCL8. Using an adenovirus to overexpress RCAN1-4, we found that RCAN1-4 is a negative regulator of CXCL8 expression in endometrial adenocarcinoma cells. Taken together our data have elucidated the molecular and cellular mechanism whereby PGF_2α_ regulates CXCL8 expression via the FP receptor in endometrial adenocarcinomas and have highlighted RCAN1-4 as a negative regulator of CXCL8 expression which may be exploited therapeutically to inhibit CXCL8-mediated tumour development.

## 1 Introduction

The human endometrium undergoes a state of inflammation and repair in a cyclical nature every 28 days [1]. This is characterized by the induction of inflammatory enzymes such as cyclooxygenase-2 (COX-2), release of prostaglandins (PG) and cytokines [2]. Chronic inflammation has been linked to increased cancer risk, for example hepatitis and colitis have been shown to promote liver [2,3] and colon cancer [2,4] respectively. By contrast, non-steroidal anti-inflammatory drug use and suppression of the COX-prostaglandin axis is associated with an inverse risk of developing cancer. In the Western world, endometrial cancer is one of the leading gynecological malignancies [5–7]. We and others have demonstrated elevated expression of the inflammatory enzyme COX-2, prostaglandin receptors and elevated signaling of the pro-inflammatory prostaglandins PGE_2_ and PGF_2α_ and growth factors such as vascular endothelial growth factor and fibroblast growth factor in endometrial adenocarcinomas [8–13]. These findings suggest that endometrial cancers are controlled in an autocrine/paracrine manner by the COX-PG axis via the synthesis and release of potent growth factors.

Dysregulated chemoattractive cytokine (chemokine) expression is known to contribute to pathologies by promoting uncontrolled proliferation, invasion and migration of cells [14]. For example the chemokine C-X-C motif ligand 1 (CXCL1) and its G protein-coupled receptor CXCR2 has been demonstrated in regulating growth and development of colon carcinomas via the COX-PGE_2_ signaling axis [15]. Furthermore we have recently shown that CXCL1 expression is elevated in endometrial adenocarcinomas and promotes neutrophil chemotaxis via PGF_2α_-FP receptor signaling to extracellular signal-regulated kinase [16]. Another chemokine ligand of the CXCR2 receptor, CXCL8, has also been implicated in tumourigenesis by enhancing melanoma cell proliferation [17], alveolar epithelial neoplasia [18] and breast cancer development [19], however its expression and role in endometrial adenocarcinoma is unclear.

Here we identified CXCL8 as a target for PGF_2α_-FP receptor signaling in endometrial adenocarcinoma cells. Using an *in vitro* model system, endometrial adenocarcinoma explants and a nude mouse xenograft model, we elucidated the molecular mechanisms mediating PGF_2α_-FP receptor signaling to CXCL8 in endometrial adenocarcinoma cells its potential role in endometrial tumourigenesis.

## 2 Materials and methods

### 2.1 Reagents

YM-254890 was kindly donated by Astellas Pharma Inc (Tsukuba, Japan). NFκB SN-50 inhibitory peptide was purchased from Biomol (Exeter, UK). Cyclosporin A and Inhibitor of NFAT-Calcineurin Association-6 (Inca-6), U73122, 4-cyano-3-methylisoquinoline (4C3MQ), SB225002 and RO-318220 were purchased from Calbiochem (Nottingham, UK). PGF_2α_, AL8810, transretinoic acid and EGTA were purchased from Sigma (Dorset, UK). The TAM-67 (dn cJun) was a kind gift from Dr Michael Birrer (National Cancer Institute, Maryland, USA) as described [20]. The CXCL8 constructs [21,22] were a kind gift from Dr Allan Brasier (Department of Internal Medicine, University of Texas Medical Branch, Galveston, Texas).

### 2.2 Cell line, culture and treatments

Wild type Ishikawa cells and Ishikawa cells engineered to stably express the full length human FP (PTGFR, accession no: NM_000959) receptor to the levels observed in endometrial adenocarcinomas, referred to as Ishikawa FPS cells, were cultured as described previously [10]. Ishikawa FPS cells were manufactured commercially, clonally selected and verified as described in our previous study [10]. Using this *in vitro* model system, we have previously demonstrated that the PGF_2α_-FP receptor signalling in Ishikawa FPS cells parallels the *ex vivo* effects of PGF_2α_ on endometrial adenocarcinoma explants [9,10]. Transient transfections were performed using Superfect (Qiagen, Crawley, UK) as per the manufacturer's protocol. The concentrations of all chemical inhibitors and antibodies were determined empirically by titration using the manufacturer's guidelines as described in our previous studies [23]. A list of all chemical inhibitors and their targets is outlined in Table 1. Cell viability was determined for each inhibitor using the CellTitre 96 AQueous One Solution assay (Promega, Southampton, UK) as described [24,25]. Cells were treated with 100 nM PGF_2α_ alone or in the presence of AL8810, YM254890, U73122 , 43CMQ, RO-318220, Cyclosporine A, Inca-6, EGTA, SN-50 for the time indicated. In parallel cells treated with vehicle (either distilled water, ethanol or dimethyl sulfoxide v/v) and chemical inhibitor served as a control for each treatment. Fold increase was calculated by dividing the values obtained from the PGF_2α_/PGF_2α_ -inhibitor treatments by the vehicle/vehicle-inhibitor treatments. All *in vitro* cell culture experiments were carried out in duplicate.

### 2.3 Patients and tissue collection

Endometrial adenocarcinoma tissue (*N* = 30; 10 of each poorly, moderately and well differentiated adenocarcinoma) and normal endometrial tissue (*N* = 30; 10 of each proliferative, early secretory and mid secretory endometrium) were obtained from women undergoing hysterectomy as detailed in our prior studies [10,24]. Ethical approval was obtained from Lothian Research Ethics Committee and written informed consent was obtained from all subjects before tissue collection.

### 2.4 Cytokine array

The human cytokine antibody array I (RayBiotech, Inc., Norcross, GA) was used according to the manufacturer's instructions using conditioned medium collected from cultured Ishikawa FPS cells treated with vehicle or 100 nM PGF_2α_ for 24 h [9]. The membranes were exposed to X-ray film and proteins quantified by densitometric analysis using the ImageQuant TL software (GE Healthcare, Little Chalfont, UK).

### 2.5 Immunohistochemistry

Immunohistochemistry was performed on endometrial adenocarcinoma tissues or nude mouse xenografts (*N* = 20) [8,9,24] using the Vision Biosystems Bond Immunostaining Robot under normal operating conditions (Leica Microsystems Wetzlar, Germany). Immunostaining was performed following antigen retrieval using specific primary antibodies CXCL8 (1:200) or mouse monoclonal BrdU antibody (1:1000) or cytokeratin 18 antibody (1:500). Control tissue was incubated with immunoglobulin (IgG) from the host species (data not shown).

### 2.6 Taqman quantitative RT-PCR

Quantitative RT-PCR was performed as detailed before [24] using sequence-specific primers and probes. Data were analyzed and processed using Sequence Detector v1.6.3 (Applied Biosystems). Expression of analyzed genes was normalised to RNA loading for each sample using the 18S ribosomal RNA or GAPDH as an internal standard. Results are expressed as fold increase above vehicle treated cells. Data are presented as mean ± SEM.

### 2.7 Secreted CXCL8 quantification

Secreted CXCL8 was quantified using an in-house enzyme linked immunosorbent assay (ELISA) described previously [26]. A matched pair of capture and biotinylated labeled detection antibodies for CXCL8 and recombinant CXCL8 were used (R&D Systems, Oxford, UK). Data are presented as mean ± SEM from at least 3 independent experiments.

### 2.8 Luciferase reporter assay

pNFκB-Luc and pAP1-Luc vectors were purchased from Clontech (Mountain View, CA). RCAN1-4 promoter reporter plasmids and pNFAT-Luc were kindly donated by Dr Takashi Minami (University of Tokyo, Tokyo, Japan) [27]. The CXCL8 promoter reporter constructs used have been previously described [22]. Cell transfection, stimulation and luciferase reporter assay was performed as described previously using the dual luciferase assay kit (Promega, Southampton, UK) [10,23]. Experiments were normalised for cell number and transfection efficiency by co-transfecting Ishikawa FPS cells with the appropriate firefly luciferase reporter together with the renilla luciferase reporter gene. Data are presented as mean ± SEM from at least 3 independent experiments.

### 2.9 Immunoblot analysis

Western blot analysis on FPS cells was carried out as described previously [25]. After resolving and immunoblotting, membranes were incubated overnight at 4°C, with a rabbit anti RCAN-1-4 antibody (1:5000), a kind gift from Dr Erik W. Bush (Myogen, Inc, Westminster, CO), together with a mouse anti β-actin antibody (1:800) (Santa Cruz Biotechnology, Santa Cruz, CA). The following day, cells were washed and incubated with goat anti-rabbit Alexafluor 680 (1:5000; Invitrogen) and goat anti-mouse IRDye™ 800 (1:5000; Rockland, Gilbersville, PA) for 60 min at room temperature. Blots were visualized using an Odyssey infrared imaging system (LI-COR, Cambridge, UK).

### 2.10 RCAN adenovirus infection

Ishikawa FPS cells were plated in 6 well plates at a density of 200,000 cells/well. After 24 h incubation, cells were washed with PBS and 1 ml of fresh medium containing 5 adenovirus molecules of infection (MOI or pfu)/plated) per cell was added to each well [25]. Cells were incubated for another 24 h and serum starved overnight before treatment with vehicle or 100 nM PGF_2α_. Adenovirus containing a scrambled sequence (CCGGCAAC AAGAT GAAGAGCACCAAC TCGAGTTGG TGCTC TTCATCTTG TTG TTTTT) was used as a control. Data are presented as mean ± SEM from at least 3 independent experiments.

### 2.11 Lentivirus shRNA gene silencing

A short hairpin RNA (shRNA) lentivirus [25] was used to knock down the expression of RCAN1. Briefly, Ishikawa FPS cells were plated in 12 well plates at a density of 80,000 cells/well with either RCAN1 ShRNA lentivirus or lentivirus containing a scrambled sequence (CCGGCAAC AAGAT GAAGAGCA CCAAC TCGAGTTGG TGCTC TTCATCTTG TTG TTTTT). After 24 h incubation, cells were infected with virus-containing media at a 1:10 dilution of virus to target cell media and 0.6 μg/ml Polybrene to give an infection rate of 1 virus per cell (1 molecule of infection; MOI). The day after, medium was replaced with fresh serum-containing medium and 48 h post-infection, the cells which were serum starved overnight, were treated with vehicle or 1 nM PGF_2α_. Data are presented as mean ± SEM from 3 independent experiments.

### 2.12 Proliferation assay

Proliferation of Ishikawa FPS cells was determined using a CellTitre 96^®^AQueous One Solution cell proliferation assay (Promega) as per the manufacturer's protocol as detailed previously [24]. Cells were treated with serum-free medium containing 5 μg/ml CXCL8 in the presence or absence of the CXCR2 antagonist SB225002 for 24, 48 or 72 h. Control wells received the same concentration of vehicle alone or vehicle and inhibitor. Following treatment, proliferation was measured by addition of the CellTitre 96®AQueous One Solution reagent as per the manufacturer's protocol. Data are presented as mean ± SEM from 3 independent experiments.

### 2.13 Xenograft tumour growth

A suspension of 5 × 10^6^ Wild type Ishikawa or Ishikawa FPS cells in a total volume of 0.2 ml DMEM was injected subcutaneously into each dorsal flank of CD1-Foxn1^nu^ mice (Charles River, UK). The mice (n = 30) were divided into two groups of equal tumour size after engraftment (1 week). The mice were injected twice weekly with 100 μg IgG or CXCL8 neutralising antibody (R&D systems, Abingdon, UK) via intra-peritoneal injection for 4 weeks. At the end of the study animals were injected with 50 mg/kg BrdU 2 h prior to culling. A proportion of the tumour was fixed in 0.2% paraformaldehyde for wax-embedding and immunohistochemistry. The animals were maintained under sterile conditions in individually vented cages. All animal care and experimental protocols were approved by the animal ethics committee of the University of Edinburgh and the Home Office of the United Kingdom government.

### 2.14 BrdU/cytokeratin staining and quantification

The expression of BrdU and cytokeratin in Ishikawa FPS IgG control and FPS CXCL8-neutralised tumours was determined by standard immunohistochemistry techniques as described above and quantified using standard stereology techniques. Briefly images were captured using a × 40 plan apo objective from a BH2 microscope (Olympus, Tokyo, Japan) fitted with an automatic stage (Prior Scientific Instruments Ltd., Cambridge, UK) using a video camera (HV-C20; Hitachi, Tokyo, Japan) and were analyzed with Image-Pro Plus 4.5.1 software with a Stereology 5.0 plug-in (Media Cybernetics, Wokingham, Berkshire, UK). A total of 100 randomised fields of view were examined and counted from 8 tumours in each group. The total number of BrdU/cytokeratin positive epithelial cells per field of view were counted and expressed as a percentage of the total points per field of view and presented as mean ± SEM.

### 2.15 Statistical analysis

The data in this study was analyzed by T-test or ANOVA using Prism 4.0c (Graph Pad, San Diego, CA).

## 3 Results

### 3.1 PGF_2α_-FP receptor activation induces CXCL8 expression and release in Ishikawa FPS cells

**Fig. 1:**
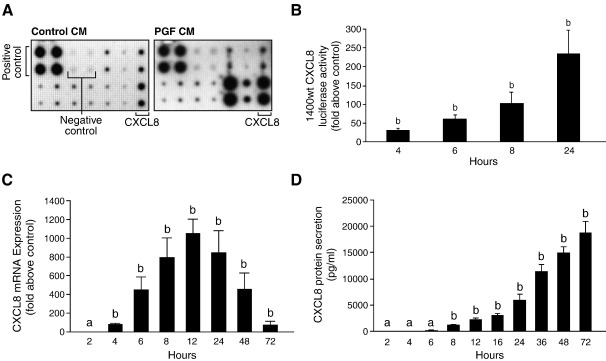
CXCL8 is induced by PGF_2α_-FP receptor signalling. (A) Chemokine release in response to 100 nM PGF_2α_ treatment in Ishikawa FPS cells. Conditioned medium obtained from Ishikawa FPS cells treated with vehicle or 100 nM PGF_2α_ for 24 h was tested for the expression of chemokines using the human cytokine antibody array (RayBiotech). (B) FPS cells were transiently transfected with the full length − 1400/+ 44 CXCL8 promoter and treated with vehicle or 100 nM PGF_2α_ for 4, 6, 8 and 24 h. CXCL8 promoter activity was determined by luciferase reporter assay. (C) CXCL8 mRNA expression in Ishikawa FPS cells following treatment of cells for 2, 4, 6, 8, 12, 24, 48 and 72 h with 100 nM PGF_2α_. (D) CXCL8 protein secretion in Ishikawa FPS cells following treatment of cells for 2, 4, 6, 8, 12, 16, 24, 36, 48 and 72 h with 100 nM PGF_2α_. (b is significantly different from a; *P* < 0.01). Data are represented as mean ± SEM.

**Fig. 2:**
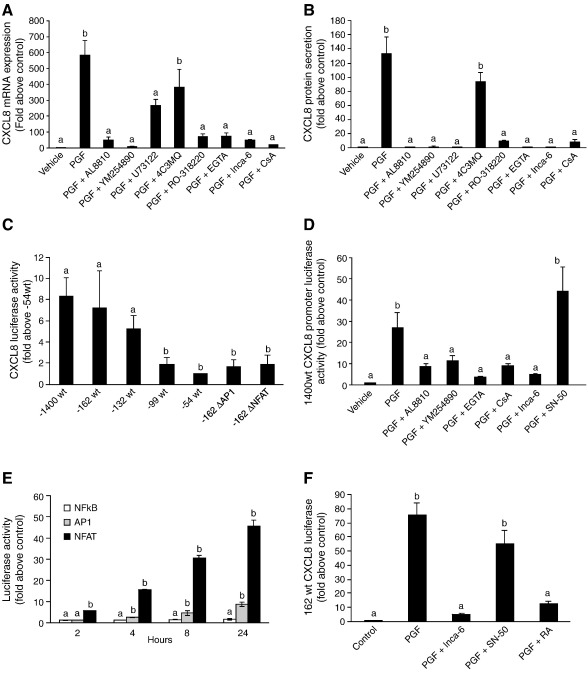
PGF_2α_-FP receptor-mediated CXCL8 is regulated via the calcium–calcineurin–NFAT pathway. (A) CXCL8 mRNA expression and (B) protein secretion in Ishikawa FPS cells treated for 8 h with vehicle, 100 nM PGF_2α_, 100 nM PGF_2α_ in the absence/presence of AL8810 (50 μM), YM254890 (1 μM), U73122 (10 μM), 4C3MQ (1 μM), RO-318220 (1 μM ), EGTA (1.5 mM), Inca-6 (40 μM) or CsA (1 μM) as determined by quantitative RT-PCR analysis and ELISA respectively. (C) CXCL8 luciferase activity in Ishikawa FPS cells transiently transfected with the full length (− 1400/+ 44) or sequential 5′ deletions (− 162/+ 44, − 132/+ 44, − 66/+ 44, − 54/+ 44) of the CXCL8 promoter or the − 162/+ 44 construct containing site-directed mutations in the NFAT or AP1 binding sites. Cells were treated with vehicle or 100 nM PGF_2α_ for 8 h and expressed as fold above the − 54/+ 44 construct which contains only the TATA box. (D) FPS cells were transiently transfected with the full length − 1400/+ 44 CXCL8 promoter and treated with vehicle or 100 nM PGF_2α_ for 8 h in the absence/presence of AL8810, YM254890, EGTA, CsA, Inca-6 or SN-50. CXCL8 promoter activity was determined by luciferase reporter assay. (E) FPS cells were transiently transfected with pAP1-luc, pNFAT-luc or pNF NFκB -luc reporter cDNA constructs and treated for 2, 4, 8 or 24 h with vehicle or 100 nM PGF_2α_. (F) CXCL8 luciferase activity in FPS cells transfected with the − 162/+ 44 CXCL8 luciferase reporter plasmid. Cells were treated with vehicle or 100 nM PGF_2α_ for 8 h in absence/presence of Inca-6, SN-50 or transretinoic acid (RA). (b is significantly different from a; *P* < 0.05). Data are represented as mean ± SEM.

Using a chemokine protein array, we identified the inflammatory chemokine CXCL8 as a target for PGF_2α_-FP receptor signaling in endometrial adenocarcinoma cells (Fig. 1A). We subsequently investigated the regulation of CXCL8 via the F-prostanoid receptor in endometrial adenocarcinoma cells and the potential role of CXCL8 in endometrial adenocarcinomas. PGF_2α_ stimulation of Ishikawa FPS cells [10] resulted in a significant time-dependent increase in the expression of CXCL8 promoter (Fig. 1B; *P* < 0.001), mRNA (Fig. 1C; *P* < 0.001) and protein (Fig. 1D; *P* < 0.001). Co-treatment of Ishikawa FPS cells with 100 nM PGF_2α_ and AL8810, YM254890, U73122, RO-318220, EGTA, Inca-6 or CsA, but not 4C3MQ significantly inhibited the expression of CXCL8 mRNA (Fig. 2A; *P* < 0.05) and release of CXCL8 protein (Fig. 2B; *P* < 0.001).

### 3.2 PGF_2α_ induces activation of CXCL8 promoter through AP1 and NFAT

To determine the regions of the CXCL8 promoter involved in regulating gene transcription in response to PGF_2α_-FP receptor interaction, Ishikawa FPS cells were transiently transfected with plasmid cDNA containing the full length (− 1400/+ 44) or serial deletions of the CXCL8 promoter (− 162/+ 44, − 132/+ 44, − 99/+ 44 and − 54/+ 44) linked to the luciferase reporter gene [21,22]. We found a considerable reduction in promoter activity when the CXCL8 promoter was truncated down to − 99 nucleotides of the 5′ flanking region of the CXCL8 gene (Fig. 2C; *P* < 0.01). No further reduction in promoter activity was observed in the − 54 nucleotides truncated construct which only contains the CXCL8 TATA box (Fig. 2C; *P* < 0.01). The use of site directed mutated versions of the − 162 CXCL8 promoter-luciferase construct at consensus binding sequences for activator protein 1 (AP1) and nuclear factor of activated T-cells (NFAT) showed that these binding sites are required for the induction of transcription of CXCL8 by PGF_2α_ (Fig. 2C, *P* < 0.01). Furthermore, we confirmed that CXCL8 transcriptional activation in response to PGF_2α_-FP receptor signalling occurred via the PKC–calcium–calcineurin–NFAT pathway (Fig. 2D, *P* < 0.01) and this was independent of nuclear factor (NF)κB (as NFAT and NFκB have similar consensus motifs on the DNA binding domain) since the NFκB inhibitory peptide SN-50 (100 μg/ml) did not reduce the activity of CXCL8 promoter induced by PGF_2α_ (Fig. 2D, *P* < 0.01). In agreement with this, Ishikawa FPS cells transfected with the pNFκB-Luc vector and treated with 100 nM PGF_2α_ did not induce NFκB-driven luciferase activity, whereas transfection of Ishikawa FPS cells with the pNFAT-Luc or pAP1-Luc vector which contains the a cis-acting NFAT or AP1 enhancer element showed a time dependent increase in luciferase activity in response to treatment with 100 nM PGF_2α_ (Fig. 2E, *P* < 0.01). We confirmed that the PGF_2α_-mediated activation of the − 162/+ 44 CXCL8 reporter gene construct occurred via the NFAT and AP1, but not NFκB, elements using specific inhibitors of NFAT (Inca-6) and AP1 (transretinoic acid; RA) and NFκB (SN-50) (Fig. 2F, *P* < 0.01).

### 3.3 NFAT and AP1 cis-enhancer elements are co-regulated by PGF_2α_

**Fig. 3:**
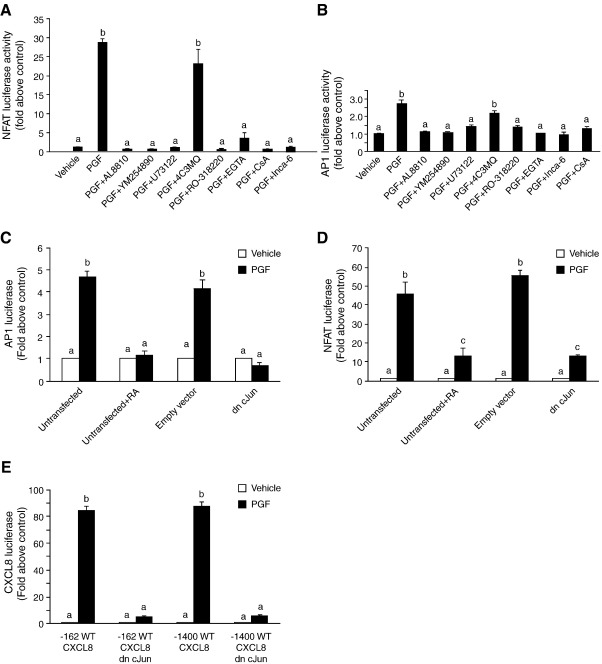
The AP1 protein c-Jun activates the AP1 and NFAT cis-acting enhancer elements to transactivate the CXCL8 promoter. FPS cells were transiently transfected with pNFAT-luc (A) or pAP1-luc (B) and treated for 8 h with vehicle, 100 nM PGF_2α_, 100 nM PGF_2α_ in the absence/presence of AL8810, YM254890, U73122, 4C3MQ, RO-318220, EGTA, Inca-6 or CsA and NFAT cis-acting enhancer DNA activation was measured by luciferase reporter assay. Ishikawa FPS cells were transiently transfected with the pAP1-luc (C) or pNFAT-luc (D) reporter plasmid and either incubated with transretinoic acid or co-transfected with an empty vector or dn c-Jun cDNA construct. Ishikawa FPS cells were transfected with the − 162/+ 44 or − 1400/+ 44 (E) CXCL8 luciferase reporter cDNA and co-transfected with empty vector or dn c-Jun cDNA. Cells were treated with vehicle or 100 nM PGF_2α_ for 8 h. (b is significantly different from a and c is significantly different from a and b; *P* < 0.01). Data are represented as mean ± SEM.

NFAT complexes with the two AP1 subunits, cJun and cFos via direct protein-protein interactions to co-ordinate promoter activity [28–30]. Treatment of Ishikawa FPS cells with 100 nM PGF_2α_ and AL8810, YM254890, U73122, RO-318220, EGTA, Inca-6 or CsA, but not 4C3MQ significantly inhibited the activation of pNFAT-Luc (Fig. 3A; *P* < 0.01) and pAP1-Luc (Fig. 3B; *P* < 0.01) luciferase. Since these transcriptional regulatory proteins are both regulated by PGF_2α_ via the PKC–calcium–calcineurin–NFAT pathways and are essential for CXCL8 activity, we performed further studies to determine whether the CXCL8 promoter was co-operatively regulated by the NFAT-AP1 complex as has been described for other cytokines such as IL-2 [31]. We found that transretinoic acid (which causes dissociation of the AP1 complex) or co-transfection of Ishikawa FPS cells with the dn c-Jun (also called TAM67) that lacks the transactivating domain [20] abolished both the transcriptional activity of AP1 (Fig. 3C, *P* < 0.01) and NFAT (Fig. 3D; *P* < 0.01) promoter elements. Furthermore, disruption of the AP1 protein complex with the dn c-Jun construct abolished the transcriptional activity of the truncated − 162/+ 44 as well as the full length − 1400/+ 44 (Fig. 3E; *P* < 0.01) CXCL8 promoter activity in response to PGF_2α_ treatment indicating that an AP1-NFAT protein complex is essential for CXCL8 activation.

### 3.4 PGF_2α_-FP receptor activation induces the expression of the calcineurin negative modulator RCAN1

**Fig. 4:**
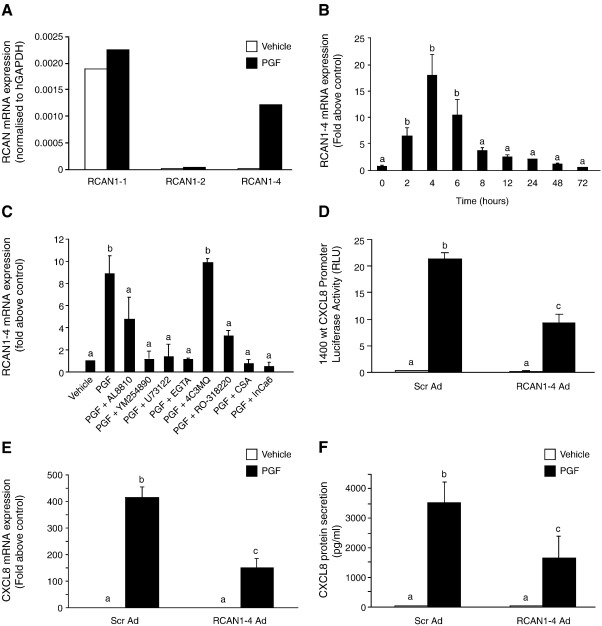
RCAN1-4 overexpression negatively regulates CXCL8 in Ishikawa FPS cells. (A) FPS cells were treated with vehicle or 100 nM PGF_2α_ for 8 h. The various RCAN isoforms (RCAN1-1, 1-2, 1-4) were detected by PCR using specific primers and normalised to expression of GAPDH. (B) RCAN1-4 mRNA expression in Ishikawa FPS cells as measured by real-time quantitative RT-PCR analysis following treatment of cells for 0, 2, 4, 6, 8, 12, 24, 48 and 72 h with 100 nM PGF_2α_. (C) FPS cells treated for 8 h with vehicle, 100 nM PGF_2α_, 100 nM PGF_2α_ in the absence/presence of AL8810, YM254890, U73122, 4C3MQ, RO-318220, EGTA, Inca-6 or CsA and RCAN1-4 expression was determined by quantitative RT-PCR analysis. Ishikawa FPS cells were transiently transfected with the full length − 1400/+ 44 CXCL8 promoter (D) or left untransfected (E and F) and infected with either scrambled control adenovirus or RCAN1-4 adenovirus for 24 h. Cells were then treated with vehicle or 100 nM PGF_2α_ for 8 h and CXCL8 promoter (D), mRNA (E) and protein (F) expression was determined by luciferase reporter assay, quantitative RT-PCR analysis or ELISA respectively (b is significantly different from a and c is significantly different from a and b; P < 0.01). Data are represented as mean ± SEM.

Having identified that the PGF_2α_-FP receptor activation of CXCL8 was mediated via the calcineurin–NFAT signalling pathway, we explored whether CXCL8 was regulated by the regulator of calcineurin 1 (RCAN1), previously known as Down syndrome critical region gene 1 (DSCR1) or Adapt 78 which is known to endogenously modulate calcineurin–NFAT signaling [32]. We identified that PGF_2α_ induces the expression of isoform 4 of RCAN1 (Fig. 4A) in rapid time-dependent manner, with maximal levels of expression after 4 h (Fig. 4B; *P* < 0.001). Co-treatment of Ishikawa FPS cells with AL8810, YM254890. U73122, RO-318220, EGTA, Inca-6 or CsA, but not 4C3MQ significantly inhibited the expression of RCAN1-4 mRNA (Fig. 4C; *P* < 0.001). These data indicate that PGF_2α_-FP receptor signalling regulates RCAN1-4 expression in a reciprocal time dependent manner to CXCL8 via the same calcium–calcineurin–NFAT signal transduction pathway regulating CXCL8.

### 3.5 RCAN1-4 overexpression inhibits PGF_2α_-induced expression of CXCL8

**Fig. 5:**
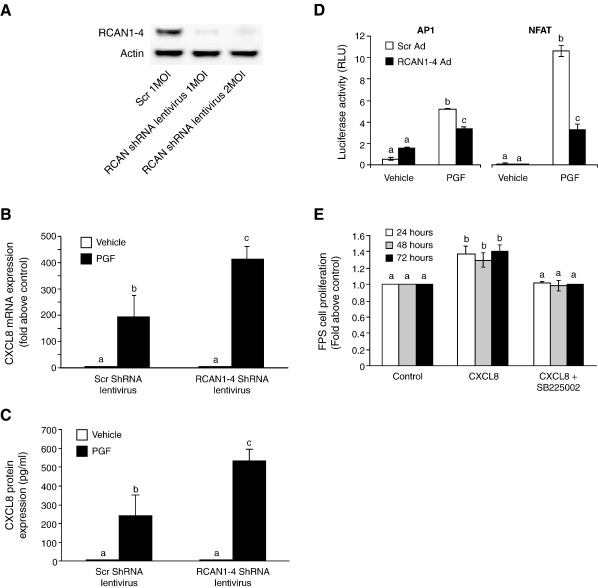
RCAN1-4 is a negative regulator of CXCL8 mRNA in FPS cells. (A) Ishikawa FPS cells were infected with 1 molecule of infection (MOI) per cell of scrambled lentivirus control or RCAN1-4 ShRNA lentivirus (1 or 2 MOI) as described in the Methods. Immunoblotting was performed using standard techniques using a specific RCAN antibody.β-Actin was used in parallel as a loading control. We confirmed that RCAN1-4 was a negative regulator of CXCL8 expression using a lentiviral ShRNA targeted against RCAN1-4. Infection of FPS cells with the RCAN1-4 lentiviral ShRNA enhanced the PGF_2α_-mediated induction of CXCL8 mRNA (B) and protein (C) expression in FPS cells treated with 1 nM PGF_2α_ for 8 h compared with control scrambled lentivirus. (D) Ishikawa FPS cells were transiently transfected with the pAP1-luc or pNFAT-luc cDNA containing the AP1 or NFAT cis-acting enhancer elements respectively and infected with either scrambled control adenovirus or RCAN1-4 adenovirus for 24 h. Cells were then treated with vehicle or 100 nM PGF_2α_ for 8 h and AP1 and NFAT luciferase activity was measured by luciferase reporter assay. (E) Cell proliferation was determined in Ishikawa FPS cells treated with vehicle or recombinant CXCL8 for 24, 48 and 72 h in the absence/presence of the CXCR2 antagonist SB225002. (b is significantly different from a and c is significantly different from a and b; *P* < 0.01). Data are represented as mean ± SEM.

RCAN1-4 is known to bind to calcineurin and inhibit activation of NFAT when overexpressed [33]. Overexpression of RCAN1-4 in Ishikawa FPS cells (Fig. 4D, E and F; *P* < 0.01) using RCAN1-4 adenovirus significantly reduced the PGF_2α_-FP receptor induction of CXCL8 promoter and mRNA expression and protein secretion compared to cells infected with the scrambled control virus. Conversely, infection of cells with RCAN1-4 lentivirus short hairpin (Sh) RNA which ablates RCAN1-4 protein expression (Fig. 5A), augmented the PGF_2α_-FP receptor activation of CXCL8 mRNA (Fig. 5B; *P* < 0.01) and protein (Fig. 5C; *P* < 0.01) confirming that RCAN1-4 is a negative regulator of CXCL8.

We further investigated the effect of disruption of NFAT activity by RCAN1-4 overexpression on activation of the NFAT and AP1 DNA transactivation domains. We found that as observed for the disruption of the AP1 transcription complex with the transretinoic acid or dn c-Jun (Fig. 3C), inhibiting NFAT activity with the RCAN1-4 adenovirus also inhibited the AP1 and NFAT (Fig. 5D; *P* < 0.01) promoter activity, giving further support for co-operativity between AP1 and NFAT.

### 3.6 CXCL8 enhances Ishikawa FPS cell proliferation *in vitro*

CXCL8 has recently been shown to enhance the proliferation and migration of squamous carcinoma cells [34]. We treated Ishikawa FPS cells *in vitro* with CXCL8 peptide and found significantly augmented cellular proliferation compared with vehicle treated cells (Fig. 5E; *P* < 0.01). Co-treatment of cells with CXCL8 and the CXCR2 antagonist SB225002 abolished the CXCL8-induced increase in cell proliferation at all time points investigated (Fig. 5E; *P* < 0.01).

### 3.7 CXCL8 expression in endometrial adenocarcinoma and normal endometrium

**Fig. 6:**
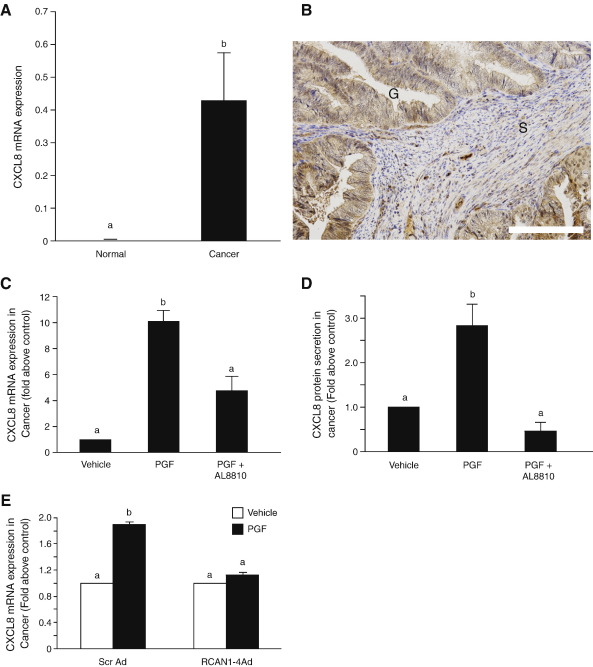
CXCL8 expression and localization in endometrial tissues. (A) Relative mRNA expression of CXCL8 in endometrial adenocarcinoma (*n* = 30; 10 of each poorly moderately and well differentiated adenocarcinoma) and normal endometrium (*n* = 30; 10 of each proliferative, early secretory and mid secretory endometrium) as determined by quantitative RT-PCR analysis. (B) Immunolocalisation of CXCL8 in an example of Well differentiated endometrial adenocarcinoma as determined by immunohistochemistry showing positive immunoreactivity (brown staining) in the glandular (G) and stromal (S) compartment. (C) CXCL8 mRNA and (D) protein expression in endometrial adenocarcinoma explants (*n* = 3) following treatment of tissue for 24 h with vehicle, 100 nM PGF_2α_ or 100 nM PGF_2α_ and AL8810. (E) Infection of endometrial adenocarcinoma explants with scrambled (Scr) or RCAN1-4 adenovirus prior to stimulation with vehicle or 100 nM PGF_2α_ for 24 h (b is significantly different from a; *P* < 0.001). Data are represented as mean ± SEM. Scale bar = 50 μm.

We next explored the expression of CXCL8 in endometrial adenocarcinoma and normal endometrial tissues and its potential regulation by PGF_2α_ via the FP receptor. CXCL8 (Fig. 6A) mRNA expression was significantly up-regulated in endometrial adenocarcinoma irrespective of grade or stage of cancer compared with normal endometrium (*P* < 0.001). CXCL8 immunoreactivity in endometrial adenocarcinomas was observed in the glandular epithelium (G; as indicated by the brown staining) with some diffuse stromal (S), staining irrespective of grade/stage of endometrial adenocarcinoma (Fig. 6B).

PGF_2α_ stimulation of endometrial adenocarcinoma explants resulted in a significant increase in the expression of CXCL8 mRNA (Fig. 6C) and secretion of CXCL8 protein (Fig. 6D), which was inhibited by co-treatment of tissue explants with the specific FP receptor antagonist AL8810 (*P* < 0.001). Furthermore, infection of endometrial adenocarcinoma explants (Fig. 6E; *P* < 0.001) using RCAN1-4 adenovirus significantly reduced the PGF_2α_-FP receptor induction of CXCL8 mRNA expression compared to tissue infected with the scrambled control virus similar to our *in vitro* data using Ishikawa FPS cells.

### 3.8 CXCL8 enhances Ishikawa FPS cell proliferation in nude mice *in vivo*

**Fig. 7:**
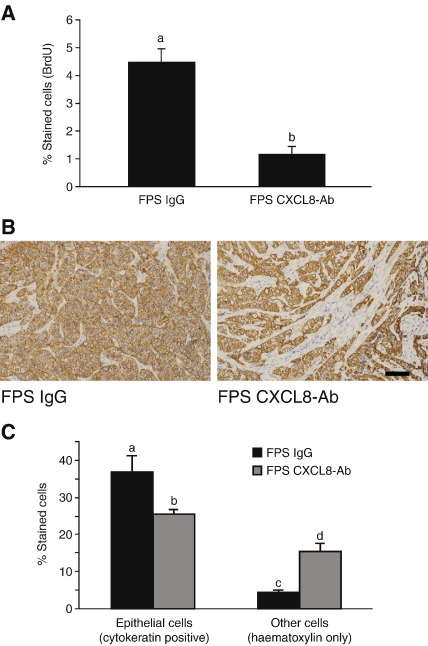
CXCL8 enhances proliferation of Ishikawa FPS cells *in vivo*. (A, B, C) The effects of CXCL8 neutralising antibody on epithelial cell proliferation *in vivo*. The CD1-Foxn1^nu^ mice were engrafted with Ishikawa FPS cells and treated with CXCL8 neutralising antibody (*n* = 10) or IgG control (*n* = 10). The incorporation of BrdU (A) in the epithelial compartment and number of epithelial cells as determined by cytokeratin 18 positive immunoreactivity (B) per tissue section were determined by immunohistochemistry (brown staining) and quantified (C) by standard stereology techniques. (b is significantly different from a; c is significantly different from a, b and d, d is significantly different from a, b and c; *P* < 0.001). Data are represented as mean ± SEM. Scale bar = 50 μm.

To explore whether CXCL8 induced by FP receptor signalling could alter tumour growth *in vivo* we injected wild type Ishikawa (WT) cells or FPS cells subcutaneously into the dorsal flanks of nude mice. Mice were then regularly injected with control IgG (WT and FPS xenografts) or CXCL8 antibody (FPS xenografts). Tumours formed from FPS cells expressed significantly higher CXCL8 mRNA as compared to WT tumours indicating that there was sufficient endogenous PGF_2α_ in nude mice to induce CXCL8 expression *in vivo* via the FP receptor similar to our observations in these cell lines vitro (data not shown). Immunohistochemical staining showed a significant reduction in the BrdU incorporation in the epithelial compartment of the mouse tumours in the animals engrafted with FPS tumour and treated with a CXCL8 neutralising antibody compared with FPS IgG controls (Fig. 7A; *P* < 0.001). In addition, we found that the administration of the CXCL8 neutralising antibody to FPS xenografts had reduced the amount of cytokeratin 18 positive immunoreactivity (as indicated by the brown staining) thereby confirming a reduction in the epithelial cell component of the xenograft tumours (Fig. 7B and C, *P* < 0.001). However, we observed no significant reduction in tumour size or volume between the various treatment groups during the 4 weeks of neutralising antibody administration, but rather a gross infiltration of other cell types (stromal, vascular, immune cells as indicated by the blue haematoxylin counterstain) to maintain tumour volume as shown in the representative image in Fig. 7B.

## 4 Discussion

Inflammation and infection are estimated to contribute to 25% of all cancer cases world wide [2, 35]. Here we demonstrate that PGF_2α_-FP receptor signalling can promote the expression of a potent chemokine with known tumourigenic and angiogenic properties [17–19].

Chemokines have emerged as important regulators of tumour function in colorectal carcinomas [15], melanomas, pancreatic, head and neck and lung carcinomas [2], and are known to promote angiogenesis and proliferation of endometrial stromal cells [14,36–38]. Moreover we have recently shown that chemokines regulated by the FP receptor can also induce neutrophil chemotaxis in endometrial adenocarcinomas [16]. In the present study we have demonstrated that PGF_2α_ promotes the synthesis and release of CXCL8 in a time-dependent manner via the protein kinase C–calcium–calcineurin–NFAT signalling pathway. Using luciferase reporter gene analysis and site directed mutations of the AP1 or NFAT [21] binding site on the CXCL8 promoter we determined that both AP1 and NFAT were essential for gene activation by PGF_2α_ and that this was independent of NFκB. Moreover we determined that both AP1- and NFAT cis-acting enhancer elements were regulated by the same protein kinase C–calcium–calcineurin–NFAT pathway which regulates CXCL8 activation. NFAT and AP1 are known to co-operate and mutually stabilise each others interaction with the DNA binding domain to allow full gene transactivation to occur, and this interaction is critical for transcription of cytokines such as interleukin-2 [39]. Furthermore, NFAT has been shown to complex with the two AP1 subunits, cJun and cFos via direct protein-protein interactions to co-ordinate promoter activity [28–30]. We found that disruption of the AP1 complex with dn c-Jun or transretinoic acid, abolished not only the ability of PGF_2α_ to mediate transactivation of the AP1 cis-acting enhancer element, but also abolished the PGF_2α_ -mediated transactivation of the NFAT luciferase reporter gene as well as the − 162 and full length − 1400 CXCL8 luciferase reporter gene. These data give further support to the co-ordinated regulation of CXCL8 transcription by a protein complex comprising of the AP1 proteins and NFAT.

NFAT activation by calcineurin, which mediates its dephosphorylation and translocation to the nucleus is known to be regulated by the regulator of calcinerin (RCAN) [32]. RCAN1-4 is known to bind to calcineurin and previous studies have shown that overexpression of this protein results in an inhibition of calcineurin activation of NFAT [27, 33]. We showed that RCAN1-4 was regulated by PGF_2α_ in a reciprocal time-dependent manner to that of CXCL8, with a peak that preceded CXCL8 by 8 h, via the calcium–calcineurin–NFAT pathway. This is in agreement with other published observations that show that expression of this isoform is induced by NFAT [27] and can negatively regulate prokineticin-prokineticin receptor 1 signaling to CXCL8 in endometrial epithelial cells [25]. Moreover adenovirus and lentivirus infection studies showed that RCAN1-4 is a negative regulator of PGF_2α_-FP receptor mediated induction of CXCL8 by inhibiting the PGF_2α_- mediated activation of the AP1 and NFAT cis-acting enhancer elements, similar to our observations for the dn c-Jun and transretinoic acid, giving further support for the co-operativity of NFAT and AP1 in mediating the full transcriptional activation of CXCL8.

We investigated the effect of CXCL8 on cellular proliferation since humanised CXCL8 antibody has recently been shown to inhibit tumour growth *in vivo* [40]. We found that CXCL8 could enhance epithelial cell proliferation via interaction with the CXCR2 *in vitro*.

To determine whether PGF_2α_-FP receptor signaling regulates CXCL8 in endometrial adenocarcinomas, we determined the expression pattern and localization of CXCL8 in endometrial adenocarcinoma tissue. We found that CXCL8 mRNA expression was elevated in endometrial adenocarcinomas irrespective of grade or stage and localized to the neoplastic glandular epithelial compartment with some diffuse stromal staining—similar to expression reported for this chemokine in normal endometrial glandular epithelium [36]. Furthermore we have shown that CXCL8 immunolocalised to the same cellular compartment in endometrial adenocarcinomas as the FP receptor and CXCR2 (the receptor for CXCL8) [16] and that CXCL8 expression and release is regulated in endometrial carcinoma explants *ex vivo* by PGF_2α_ via the FP receptor. Moreover we have shown that CXCL8 expression in endometrial adenocarcinoma explants is negatively regulated by RCAN1-4 since infection of endometrial adenocarcinoma explants with RCAN1-4 adenovirus abolished the PGF_2α_-FP receptor-mediated induction of CXCL8. These data suggest that the molecular mechanism that we have elucidated for the regulation of CXCL8 via the RCAN1-4 pathway *in vitro* can potentially regulate CXCL8 in endometrial adenocarcinomas. Although little is known of the role of CXCL8 in endometrial cancer, dysregulated CXCL8 and CXCR2 expression has recently been proposed to play a role in other endometrial disorders such as endometriosis [14, 38]. Furthermore, CXCL8 has recently been shown to enhance the proliferation and migration of squamous carcinoma cells [34] and its elevated secretion in esophageal squamous cell carcinomas has been related to lymph node and distant metastases [41].

Finally we investigated the impact of CXCL8 expression on tumour development *in vivo* by engrafting nude mice with wild type Ishikawa cells and FPS Ishikawa cells. We found that CXCL8 expression was significantly elevated in FPS xenograft tumours compared with wild type tumours indicating that FP receptor was being activated by endogenous PGF_2α_ *in vivo* similar to our observations using FPS cells and administering exogenous PGF_2α_ *in vitro*. We further confirmed that CXCL8 played a role in epithelial cell function *in vivo*, as Ishikawa FPS cell tumour xenografts in nude mice treated with a CXCL8 neutralising antibody displayed reduced neoplastic epithelial cell proliferation characterised by reduced BrdU incorporation and reduced cytokeratin 18 immunoreactivity, compared to IgG treated controls. Further studies are currently underway in our laboratory to determine whether alterations in cell type and density within a tumour effects tumour outcome.

**Fig. 8:**
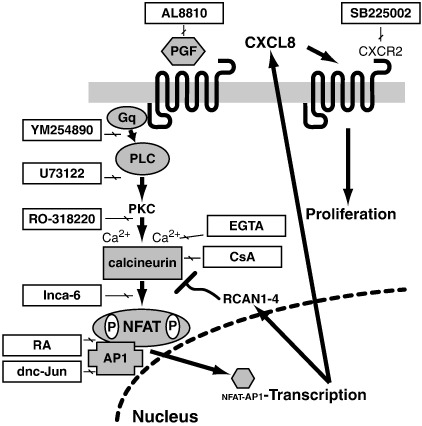
Summary. A schematic summary of our findings which includes the points of inhibition of the various inhibitors outlined in Table 1 on the signalling cascade. PGF_2α_-FP receptor activation in endometrial adenocarcinoma cells promotes the induction of RCAN1-4 and CXCL8 via the Gq-PLC-PKC–calcium–calcineurin–NFAT cascade. The expression of RCAN1-4 and CXCL8 are reciprocal and RCAN1-4 expression precedes CXCL8 expression by 4–8 h. We found that RCAN1-4 acts as a negative regulator of the calcineurin pathway to prevent over induction of CXCL8, such that when levels of RCAN1-4 are high, CXCL8 levels are suppressed. When RCAN1-4 levels wane over time, CXCL8 expression is induced to maximal to promote tumour cell proliferation via the CXCR2 receptor on epithelial cells.

Taken together, our data (as summarised in Fig. 8) show that PGF_2α_-FP receptor activation in endometrial adenocarcinoma cells promotes the activation of RCAN1-4 and CXCL8 via the Gq-PLC-PKC–calcium–calcineurin–NFAT. The activation of RCAN1-4 is reciprocal to CXCL8, such that at the peak of RCAN1-4 expression, CXCL8 expression is minimal and vice versa. As RCAN1-4 expression reduces over time, the expression of CXCL8 increases to promote tumour cell proliferation. Furthermore, we confirmed that RCAN1-4 is a potent negative regulator of CXCL8 *in vitro* and *ex vivo* in endometrial adenocarcinomas explants and that the regulation of transcriptional activation of CXCL8 by PGF_2α_ occurs via the co-operativity between AP1 and NFAT DNA binding elements. To our knowledge this represents the first report mapping the molecular and cellular regulation of CXCL8 by prostanoids and its potential involvement in endometrial cancers.

## Figures and Tables

**Table 1:** List of reagents summarising the targets of each compound.

Compound	Concentration	Target	Reference
AL8810	50 μM	FP receptor antagonist	[42]
YM254890	1 μM	Gq/11 inhibitor	[43,44]
U73122	10 μM	PLC beta inhibitor	[45]
4C3MQ	1 μM	Protein kinase A inhibitor	[46]
RO-318220	1 μM	Protein kinase C inhibitor	[47]
Inca-6	40 μM	Inhibitor of interaction between calcineurin and its substrate nuclear factor of activated T cells (NFAT); blocks at the substrate recognition site but not at the catalytic site. Inhibits NFAT dephosphorylation.	[48]
CsA	1 μM	Inhibits the dephosphorylation and translocation of NFAT to the nucleus by inhibiting calcineurin phosphatase activity	[49]
SN-50	100 μg/ml	Peptide inhibitor of NF-kB translocation to the nucleus	[50]
Trans-retinoic acid (RA)	1 μM	Inhibits AP1 complex formation (cJun homodimerisation or cJun/cFos heterodimerisation)	[51]
Ethylene glycol tetraacetic acid (EGTA)	1.5 mM	Chelating agent with higher affinity for calcium than magnesium	[52]
SB225002	30 nM	Selective non-peptide inhibitor of CXCR2, inhibiting IL-8 binding to CXCR2	[53]
